# Supplementation with Polyphenols (Olive and Grape Extracts) Improves Pregnancy Outcomes in Underfed Sheep

**DOI:** 10.3390/antiox14121489

**Published:** 2025-12-12

**Authors:** José Luis Pesántez, Paula Martínez-Ros, Antonio González-Bulnes, M. Carmen López-Mendoza, Francisco Sales, Nesrein M. Hashem, Mónica De los Reyes, Luis A. Raggi, Natalia Yeste-Vizcaíno, Víctor H. Parraguez

**Affiliations:** 1 Escuela de Medicina Veterinaria, Facultad de Ciencias Agropecuarias, Universidad de Cuenca, Cuenca 010107, Ecuador; jose.pesantez@ucuenca.edu.ec; 2 Facultad de Veterinaria, Universidad CEU Cardenal Herrera, 46115 Valencia, Spain; antonio.gonzalezbulnes@uchceu.es (A.G.-B.); clopez@uchceu.es (M.C.L.-M.); 3 Instituto de Investigaciones Agropecuarias (INIA), CRI Kampenaike, Punta Arenas 6210489, Chile; fsales@inia.cl; 4 Animal and Fish Production Department, Faculty of Agriculture, Alexandria University, Alexandria 21545, Egypt; nesreen.hashem@alexu.edu.eg; 5 Facultad de Ciencias Veterinarias y Pecuarias, Universidad de Chile, Santiago 8820808, Chile; mdlreyes@uchile.cl (M.D.l.R.); lraggi@uchile.cl (L.A.R.); 6 Faculty of Veterinary Medicine, Universidad Autónoma de Barcelona (UAB), 08193 Cerdanyola del Vallés, Spain; natalia.yeste@uab.cat; 7 Facultad de Ciencias Agronómicas, Universidad de Chile, Santiago 8820808, Chile

**Keywords:** antioxidants, undernutrition, sheep gestation, birth weight

## Abstract

Our study aimed to test the hypothesis that supplementation with a combination of dry grape extracts (DGS) and olive-originated hydroxityrosol (HxT) polyphenols during ovine gestation under moderate nutritional restriction allows for the improvement of the birth weight and size of newborn lambs, especially in more demanding pregnancies such as twins. Twenty-six pregnant ewes (fourteen ewes with singleton pregnancies and twelve ewes with twin pregnancies) were divided in two equal groups, treated or not with the polyphenols, and involved in the study. The results indicate that supplementation with the combination of polyphenols, without modifying maternal body weight and body condition score, increased the maternal total antioxidant capacity (*p* < 0.05) and, interacting with pregnancy rank, improved maternal metabolic status by reducing β-hydroxybutyrate and non-esterified fatty acids (*p* < 0.05). Afterwards, supplementation improved birth weight and size of newborns in the most compromised pregnancies (i.e., twin pregnancies; twins lambs born to supplemented ewes were significantly heavier than those born to control ewes (3.3 ± 0.13 vs. 2.8 ± 0.23 kg, respectively; *p* < 0.05).

## 1. Introduction

Sheep production is usually associated with harsh environments (either semiarid or dry areas and/or at high altitude), causing undernutrition, due to the limited capacity to produce forage in adequate quantity and quality to meet the demands of sheep flocks [1,2]. During pregnancy, maternal undernutrition or exposure to high altitudes evokes fetal hypoxemia and oxidative stress [3,4]. Both circumstances affect the adequate development of pregnancy, leading to restricted intrauterine growth and fetal re-programming [5], which are even more boosted in the case of twin pregnancies [4,5]. Antioxidant supplementation in pregnant ewes in such disadvantageous environments, has been shown to have beneficial effects by improving the birth weight of the offspring and their survival [3,6].

The European Mediterranean countries are characterized by producing and consuming significant quantities of meat and dairy products from sheep origin [7]. The so-called Mediterranean Diet also includes other products such as fish, vegetables, fruits, olive oil, and wine [7]. Recent studies suggest that olive oil and wine production will increase in response to growing global demand, a trend that will be driven not only by traditional Mediterranean producers but also by new entrants to the market [8,9]. However, the production of both olive oil and wine is controversial for the environment, due to generation of a large number of residues that are potentially polluting the surroundings [10,11]. On the other hand, such residues are, in fact, by-products which contain antioxidant polyphenolic compounds that can be extracted and used as nutritional supplements in case of risk of increased oxidative stress [12,13,14,15]. Hence, the use of antioxidants generated from olives and grapes by-products may be highly beneficial for animal production, and specifically sheep production, which would be an outstanding example of sustainability and circular economy.

In fact, polyphenolic compounds from different plant origin have been tested in different livestock species [15,16,17,18]. These compounds are currently extracted, characterized and marketed as nutritional additives. Specifically, dry grape extracts (DGS; >80% polyphenols) and hydroxityrosol (HxT; derived from olive by-products and leaf) are authorized in the European Union for their use as nutritional additives [19,20]. Thus, DGS supplementation in well-nourished sheep during the first 47 days of lactation significantly increased live weight and carcass weight of the lambs at 115 days of age [21]. Moreover, weaned lambs, consuming a balanced diet supplemented with DGS for 60 days, resulted in a significant increase in antioxidant status, daily weight gain, feed conversion efficiency, and immune status [18]. On the other hand, HxT has not been tested in sheep or other ruminants. However, in monogastric farm animals such as pigs, HxT supplementation has shown interesting results. Sows treated with HxT from day 35 of gestation showed increased antioxidant status, birth weight and size, and postnatal development of their piglets [22].

In the present study, we tested the hypothesis that supplementation with a combination of DGS and HxT during ovine gestation under moderate nutritional restriction, improves the birth weight and size of newborn lambs, especially in more demanding pregnancies such as twins.

## 2. Materials and Methods

### 2.1. Ethics, Animals, and General Management

The study was carried out at the experimental farm of the Faculty of Veterinary Medicine, CEU Cardenal Herrera University (Alfara del Patriarca, Valencia, Spain; February to July 2024) according to the European Union Directive and the Spanish Policy for Animal Protection RD53/2013.

Thirty Segureña ewes, 3–4 years old, 54.5 ± 1.7 Kg of body weight (BW) and 3.5 ± 0.2 of body condition score (BCS) in accordance with Jefferies scale [23], were used. The animals were kept in a common, roofed paddock (160 m^2^), with daily access to an open paddock (700 m^2^), receiving alfalfa hay, oat straw, and water *ad libitum*. Ewes were subjected for estrous synchronization using progesterone intrauterine devices (CIDR OVIS^®^, 0.35 g progesterone; Zoetis, Spain), for 12 days. After devices removal, ewes were hand-mated with three rams of proven fertility.

Forty days after mating, pregnancy and number of embryos were determined by transrectal ultrasonography (M-Turbo, FUJIFILM Sonosite, Inc., 21919 30^th^ Drive SE Bothell, WA 98021, USA). Twenty-six ewes became pregnant, and fourteen ewes with singleton pregnancies and twelve ewes with twin pregnancies were involved in the study. Immediately after the ultrasound examination and until lambing, these ewes were separated by gestation rank (singleton and twin) in adjacent paddocks and subjected to a permanent regime of moderate nutritional restriction (~20% energy/protein restriction), according to the differential requirements by stage of gestation and rank [24]. Diets consisted of alfalfa hay and oat straw in amounts adjusted to provide protein and energy meeting approximately 80% of the daily requirements of ewes carrying single (82 g CP; 8.4 MJ/kg ME) or twin fetuses (99 g CP; 10.3 MJ/kg ME) until day 90 of gestation. From day 91 until parturition, daily dietary allowances were increased to 108 g CP and 10.4 MJ/kg ME for single-bearing ewes, and to 132 g CP and 13.2 MJ/kg ME for twin-bearing ewes. Each group of gestational rank was divided into two equal treatment groups, half supplemented with antioxidants (AOx), and half controls without AOx. Hence, four groups were finally considered as follows: single pregnancies supplemented with AOx (n = 7); single control pregnancies (n = 7); twin pregnancies supplemented with AOx (n = 6); and twin control pregnancies (n = 6). From the start of treatment until the end of gestation, weight and BCS of each ewe were biweekly assessed. The allocation of treatment (AOx or control) within each gestational rank was carried out using block randomization based on ewe body weight.

AOx supplementation, started simultaneously with the nutritional restriction. It was performed with DGS (0.8 mg Kg^−1^ ewe live weight, Nuxafen^®^, Alvinesa, Spain) and HxT (0.01 mg Kg^−1^ ewe live weight, Olive Fruit Extract liquid^®^, Genosa, Alvinesa, Spain). Since no information was available on the combined use of both products in ruminants, the doses used in this study were estimated according to the following criteria. For DGS, we consider its antioxidant power to be equivalent to 10 times that of pure vitamin E, as indicated by the manufacturers. Additionally, improvements in antioxidant status and lambs birth weight (mainly twins) was observed when pregnant ewes received a daily dose of 7–8 mg Kg^−1^ body weight of vitamin E [25], suggesting that 0.8 mg Kg^−1^ of DGS could mimic the antioxidant capacity achieved with vitamin E. In the case of HxT, no studies on supplementation in small ruminants during gestation were found. According to studies in pregnant sows under nutritional restriction, an approximate daily dose of 0.02 mg Kg^−1^ body weight, supplemented during the last trimester of gestation, significantly increased the antioxidant status and birth weight of piglets [22]. Therefore, to enhance the effect of DGS, we decided to include a dose of 0.01 mg Kg^−1^ body weight of HxT. Both products were mixed and suspended in physiological solution to prepare and administrate a dosage of 1 mL of suspension for each 10 kg of ewe’s body weight. The volume of solution, adjusted to the body weight of each pregnant ewe, was daily administered directly into the mouth of each treated animal with a syringe and, in order to maintain the same management, the control ewes received orally an equivalent volume of physiological solution. The dose administered was adjusted biweekly according to the body weight of each animal. One week before the estimated date of delivery, blood samples (5 mL) were taken with heparinized syringes from the jugular vein of the ewes for evaluation of antioxidant status and biochemical parameters. The blood was centrifuged at 1300× *g* for 5 min and the plasma obtained was stored at −80 °C until analysis. At delivery, both nutritional restriction and AOx or physiological solution supplementation were suspended and the ewes returned to their full diet.

Approximately 4 h after delivery, the newborn lambs were assessed for sex, birth weight, and body size (body length, fore- and hindlimb length, and thoracic and abdominal circumference). Simultaneously, a colostrum sample (20 mL) was collected from each ewe, immediately labeled, and stored at −20 °C until compositional analysis.

### 2.2. Analysis of Blood and Colostrum Samples

Oxidative stress in blood plasma samples was evaluated through measurement of total antioxidant capacity (TAC) and malondialdehyde (MDA, a lipid oxidation biomarker) by enzyme immunoassay (ELISA). TAC was measured in 10 µL of blood plasma using the Antioxidant Assay Kit^®^ (Cayman Chemical Company, Ann Arbor, MI, USA). MDA was measured in 100 µL of blood plasma using the TBARS Assay Kit (Cayman Chemical Company, Ann Arbor, MI, USA). Absorbance for MDA and TAC were assessed in a microplate reader (FLUOstar Omega BMG LABTECH GmbH, Ortenberg, Germany) at 540 and 405 nm, respectively. All the assays were performed in duplicate in accordance with the supplier instructions and has been previously adapted and tested for sheep [3,5].

Blood biochemistry analysis consisted of the measurement of β-hydroxybutyrate (BHB), glucose, lactate, non-esterified fatty acids (NEFAs), triglycerides, protein, and urea, using a clinical analyzer (Konelab 20i, Thermo Fisher Scientific, Madrid, Spain).

The colostrum samples were diluted in deionized distilled water (1:1 *v*/*v*), quickly homogenized and assayed at 23 °C for evaluation of the fat, protein, lactose, total solids, and non-fat solids content using an IR spectrophotometer (MilkoScan ™ 7RM, Foss Iberia, Barcelona, Spain).

### 2.3. Statistical Analysis

Data were checked for normality and homoscedasticity and transformed when necessary. For traits measured once, a 2 × 2 factorial design was used with pregnancy rank (single vs. twin) and treatment (Control vs. AOx) as fixed factors. Maternal and newborn traits were analyzed by two-way analysis of variance using the general linear model (GLM) procedure from the GraphPad Prism 10 statistical software (GraphPad Software, Inc., Boston, MA, USA) to evaluate the main effects of pregnancy rank and treatment. No interaction between factors was observed for any of the variables analyzed; therefore, interaction terms are not included in the tables. Maternal BW and BCS, which were measured repeatedly over time, were analyzed separately from the factorial model. Because the biological interest was to characterize temporal patterns within each pregnancy rank, longitudinal changes in these variables were evaluated using repeated-measures ANOVA conducted independently for single- and twin-bearing ewes. To complement this analysis, mean differences between pregnancy ranks at each gestational time point were assessed using independent-samples t-tests (cross-sectional comparisons). A probability of *p* ≤ 0.05 was considered statistically significant. Results are expressed as mean ± SEM.

## 3. Results

### 3.1. Maternal Traits

#### 3.1.1. Changes in Maternal BW and BCS over Time of Pregnancy

**Figure 1:**
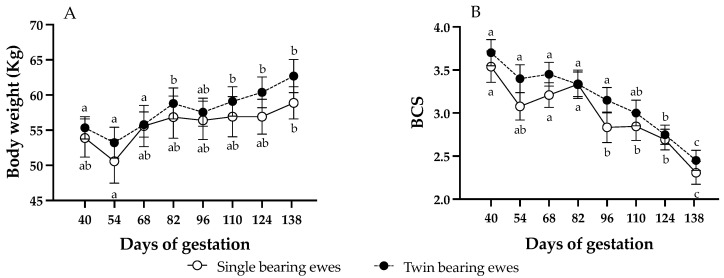
Changes (mean ± SEM) in ewe’s body weight (**panel A**) and BCS (**panel B**) throughout pregnancy. Different letters within each pregnancy rank indicate significant differences between gestation times (*p* < 0.05; longitudinal comparisons). No differences were detected in body weight or BCS between ewes with single or twin fetuses at each time point (cross-sectional comparisons).

Body weight and BCS were not affected by the AOx treatment or by the gestational rank (*p* > 0.05). Therefore, no differences in mean BW (single bearing ewes, control: 57.1 ± 0.8, AOx: 55.8 ± 0.9; twin-bearing ewes, control: 57.6 ± 0.7, AOx: 59.1 ± 1.3; *p* = 0.062) or BCS (single bearing ewes, control: 3.1 ± 0.1, AOx: 2.8 ± 0.1; twin-bearing ewes, control: 3.2.6 ± 0.2, AOx: 3.1 ± 0.1; *p* = 0.30) between groups were detected. Then, Figure 1 presents them as twin and singleton pregnancies, without differentiating between those supplemented with AOx or not supplemented. Body weight of the ewes increased progressively over time of pregnancy, without significant differences between pregnancy ranks. Ewes carrying singletons gained around 5.3 kg during gestation, with significant differences only between days 54 and 138; meanwhile ewes carrying twins gained around 7.4 kg, showing significant increases from day 82 of gestation. Conversely, BCS showed an inverse pattern, with a marked decrease along gestation and again without significant differences between pregnancy ranks, being significantly lower from day 96 of gestation in singleton ewes and from day 124 in twin ewes. Ewes carrying singletons decreased BCS by 1.2 points, while those with twins by 1.3 points but without significant differences.

#### 3.1.2. Assessment of Maternal Antioxidant/Oxidative Status at 140 Days of Pregnancy

**Figure 2:**
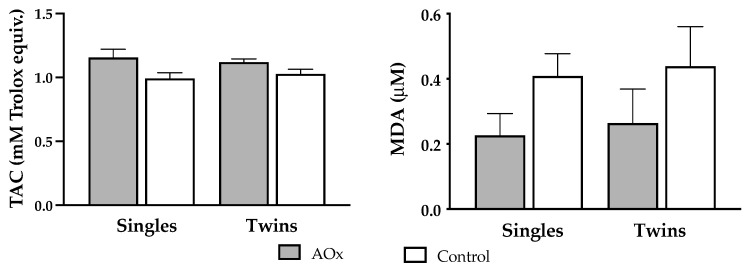
Plasma levels (mean ± SEM) of biomarkers of antioxidant/oxidative status. Left graph depicts total antioxidant capacity (TAC; Rank *p* = 0.995, AOx *p* = 0.004) and right graph depicts malondialdehyde (MDA; Rank *p* = 0.212, AOx *p* = 0.443) concentrations at 140 days of gestation, in ewes carrying singleton and twin pregnancies and supplemented or not with AOx. There was no interaction between the factors in any of the two variables.

Supplementation with antioxidants induced a significant increase (*p* = 0.004) in plasma TAC at 140 days of pregnancy in both single and twin pregnant ewes (Figure 2). Conversely, concentrations of MDA were lowered in singleton and twin pregnant sheep receiving antioxidants supplementation, but the difference did not reach significance.

#### 3.1.3. Assessment of Maternal Metabolic Biomarkers

There were no major changes in plasma biomarkers of metabolic status at 140 days of pregnancy, excepting BHB and NEFAs which were affected by pregnancy rank (Table 1), with twin-bearing control ewes showing the highest values. Additionally, twin-bearing ewes showed a marked trend towards higher blood lactate concentrations compared to those carrying singletons.

#### 3.1.4. Assessment of Colostrum Composition

There were no effects from either number of fetuses or antioxidant supplementation on the colostrum nutritional composition (Table 2).

### 3.2. Newborn Traits

#### 3.2.1. Birth Weight

**Figure 3:**
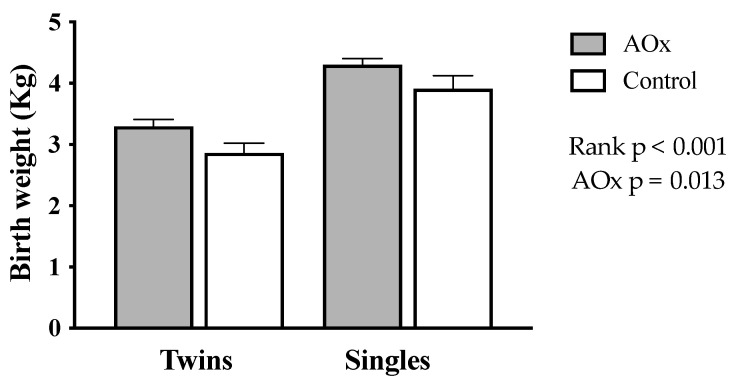
Body weight at birth from lamb’s born to single- and twin-bearing ewes supplemented or not with AOx.

Birth weight of the lambs is shown in Figure 3. It was significantly higher in singleton than in twin lambs (4.0 *±* 0.15 vs. 3.1 *±* 0.11 Kg, respectively; *p* < 0.001). The supplementation with antioxidants also increases the birth weight of lambs (*p* = 0.013), an effect that was greater in twins (AOx: 3.3 *±* 0.13 Kg, control: 2.8 *±* 0.23 Kg) than singles (AOx: 4.1 *±* 0.21 Kg, control: 3.9 *±* 0.22 Kg).

#### 3.2.2. Body Size at Birth

Body measurements of newborn lambs are reported in Table 3. The body-length and the thoracic circumference of the newborn lambs were not significantly affected by pregnancy rank or treatment, although AOx supplementation showed a trend to promote thoracic perimeter. The abdominal perimeter, on the other hand, was affected by both the pregnancy rank and the AOx supplementation and twins had a smaller abdominal perimeter than singletons, but both singleton and twins born to ewes supplemented with AOX showed highest values than their control counterparts. Finally, both forelimb and hindlimb lengths were greater in singleton lambs than in twins, with no AOx effects.

## 4. Discussion

The results of the present study support the idea that twinning in sheep under moderately challenged pregnancies (i.e., 20% nutritional restriction) is associated with maternal metabolic stress, as indicated by significantly higher plasma BHB and NEFA concentrations and numerically higher lactate levels, while differences in body weight (BW) and body condition score (BCS) were observed only during the last month of gestation. Compared with singletons, these ewes give birth to lambs with lower birth weight and smaller body size. Supplementation of underfed pregnant ewes with a combination of polyphenols (DGS and HxT) improves their antioxidant status and, without altering BW, BCS, or metabolic traits, also enhances the birth weight and size of the offspring, particularly in the most compromised pregnancies (i.e., twin pregnancies).

### 4.1. Changes in Maternal Body Weight, Body Condition, and Metabolic Status

The assessment of changes in BW and BCS throughout gestation confirmed the undernourished state of the ewes in our study, as evidenced by a smaller increase in BW compared with values typically reported for well-nourished pregnant ewes (approximately 10% of BW in singleton-bearing ewes and about 18% in twin-bearing ewes. Concomitantly, the BCS should not decrease more than 0.5 points in both singleton and twin pregnancies but, in our study, BCS decreased by 1.2–1.3 points [26]. These changes were similar in both singleton and twin pregnancies, and no significant differences in BW or BCS were detected between pregnancy ranks.

Conversely, pregnancy rank affected the metabolic status of the dams, with twin-bearing ewes showing higher plasma concentrations of BHB and NEFAs, and a trend for higher lactate concentrations, than sheep carrying singletons. BHB, NEFAs, and lactate reflect the level of lipid catabolism, and their concentration increases when glucose metabolism is deficient [27]. Thus, the higher plasma concentrations of BHB and NEFAs, along with the upward trend in lactate, reflect increased metabolic stress and impaired energy balance in twin-bearing ewes, resulting in intensified lipomobilization, as previously shown in cows [28], and similar to previous studies in both meat and dairy sheep [29]. Moreover, we have to note that both BHB and NEFAs levels in control sheep with twin pregnancies remained above physiological levels for the species (0.36–0.80 and 0.18–0.68 mmol/L, respectively) [30,31,32], which is indicative of a considerable metabolic challenge in these sheep.

In our study, supplementation with polyphenolic compounds did not affect maternal BW and condition, as previously described for other AOx therapies in pregnant sheep under more severe conditions of nutritional restriction [5]. However, it improved the antioxidant/oxidative status, as evidence by the significant increase in TAC and the reduction in MDA plasma concentrations.

The increase in TAC is a protective mechanism particularly relevant in multiple pregnancies, where oxidative stress is more pronounced [5]. This stage is characterized by the progressive rise in energy metabolism to sustain rapid fetal growth, placental function, and maternal metabolic demands [33,34]. Consequently, the elevated metabolic activity accelerates the production of reactive oxygen species (ROS) and enhances oxidative damage [35,36], which, if not adequately counteracted, may compromise cellular integrity, impair fetal development, and predispose animals to various diseases [37], ultimately affecting future productive [38] and reproductive [39] performance. Oxidative stress states, resulting from elevated ROS concentrations in ewes, have been associated with restricted fetal growth [4,35,40].

Lipid peroxidation generates a wide range of aldehydes, among which malondialdehyde (MDA) is recognized as a specific biomarker of oxidative stress [41]. The increase in oxidative stress during gestation is associated with elevated MDA concentrations [42]. To maintain redox balance, the organism relies on several endogenous defense mechanisms based on enzymatic scavenging systems, among which superoxide dismutase (SOD), catalase (CAT), and peroxidases are particularly prominent. However, under certain conditions such as gestation, these systems may be overwhelmed, leading to a state of oxidative stress. To counteract this deficit, the use of exogenous antioxidants has been shown to be an effective strategy to reinforce the organism’s defensive capacity, thereby reducing MDA levels [4].

In our study, supplementation with DGS and HxT was associated with a trend towards reduced MDA concentrations, as previously reported in other studies [5,43], although this decrease did not reach the expected level of statistical significance. This suggests that, while antioxidant supplementation contributed to strengthening endogenous systemic defenses, the degree of lipid peroxidation in these females may not have been sufficiently pronounced to allow the detection of a significant reduction with a clear effect within this period. Nevertheless, studies in dairy cows during late gestation have not revealed significant changes in MDA concentrations. This apparent absence of variation may largely be explained by the high individual variability in this biomarker, which could mask physiologically relevant changes [44]. Therefore, supplementation with polyphenolic compounds enhances the antioxidant capacity of ewes during the final third of gestation, irrespective of whether they are carrying singletons or twins. At the same time, this positive effect on oxidative status occurs without substantially altering the body reserves of the ewes.

### 4.2. Changes in Newborn Lambs’ Body Weight and Size

Maternal undernourishment is known to induce intrauterine growth restriction and, thus, low birth weight of the newborns [45], even more in case of twinning [4]. Lamb birth weight and morphometric measurements are indicators of intrauterine growth rate and can be influenced by maternal factors (age and parity), placental factors (size and blood flow), fetal factors (number and sex), and environmental conditions (nutritional status and hypobaric hypoxia) [3,46]. In our study, singleton lambs had a mean birth weight of 4.0 kg, whereas twins averaged 3.1 kg. This difference represents an approximate reduction of 0.9 kg, equivalent to around 22% less weight in twins compared with singletons, directly reflecting that multiple pregnancies increase intrauterine competition for nutrients and oxygen, thereby limiting fetal growth [4,47,48].

Previous studies have also shown that intrauterine growth restriction is related to impaired supplies of nutrients and oxygen and, consequently, a weakened antioxidant defense system [49,50,51]. Ewes under extensive rearing are prone to undernourishment and intrauterine growth restriction, mainly in case of twin pregnancies, which is associated with the occurrence of an oxidative stress condition [4]. In these sheep, the effects of intrauterine growth restriction may be alleviated by the maternal supplementation with the antioxidant vitamins C and E, which ameliorate the antioxidant/oxidative ratio, improve the placental function, and increase the weight and viability of the newborn [3,6,25]. In the present study, similarly to these previous data in sheep affected by undernourishment and/or hypoxia and treated with vitamins C and E, the use of antioxidant polyphenols (olive and grape extracts) induced a non-significant increase of around 200 g in the birth weight of singletons but a significant increase of around 500 g in the birth weight of twins.

The lack of effect of maternal antioxidant (AOx) supplementation on the birth weight of singleton lambs, indicates that in single-fetus pregnancies, under a maternal 20% food restriction, nutrient availability is sufficient to sustain optimal growth irrespective of supplementation [4]. In contrast, twin lambs born to AOx-supplemented ewes were significantly heavier compared with those born to control ewes (3.3 kg vs. 2.8 kg; *p* = 0.035). This suggests that AOx supplementation may mitigate oxidative stress or the metabolic challenges associated with multiple pregnancies, allowing for improved nutrient utilization or enhanced placental efficiency [6]. The data indicates that antioxidant support becomes particularly relevant under conditions of increased fetal competition, when the risk of intrauterine growth restriction rises as litter size increases [52] and AOx supplementation counteracts this negative effect, and could potentially improve lamb survival, which is highly correlated to lamb weight at birth [53].

Morphometric measurements not only reflect physical conformation, carcass structure, growth conditions, and the relationship of development among tissues and organs, but also provide additional information on growth patterns during gestation and postnatal development of the offspring [54]. Body length and thoracic circumference of newborn lambs were not affected by type of pregnancy or treatment, although AOx supplementation showed a tendency to promote thoracic girth. These findings are consistent with previous reports, where no marked differences in these morphometric traits were observed [3].

Abdominal circumference of lambs was influenced by both type of pregnancy and maternal supplementation. Twins presented significantly lower values compared with singletons; however, in both groups, this measure was greater when the ewes received AOx. Since abdominal circumference constitutes an indirect indicator of visceral development and gastrointestinal capacity, this finding suggests that AOx supplementation favors intra-abdominal growth, which may enhance metabolic and digestive adaptation in early life. Similarly, singleton lambs exhibited longer forelimbs and hindlimbs, a development consistent with their higher birth weight (4.0 ± 0.15 kg) compared with their twin counterparts. This pattern aligns with their greater body size and growth potential during gestation, reflecting differences in nutrient availability throughout this stage. Although AOx supplementation did not significantly influence these fetal traits, the evidence indicates that its administration during gestation does not alter the linear dimensions of the limbs but rather exerts a more direct effect on overall birth weight and other body measurements.

### 4.3. Changes in Colostrum

Colostrum composition, including fat, protein, and lactose levels may vary according to several factors, such as parity, litter size, nutrition, and potentially breed [55]. In the present study, the percentages of fat, protein, lactose, total solids, and non-fat solids remained within the physiological ranges reported for this species [55] and neither the rank, nor antioxidant supplementation, exerted a significant effect on the nutritional composition of colostrum. This contrasts with what was observed in sheep under more severe undernutrition, where those that gave birth to a single lamb have more protein and lactose than those that gave birth to twins [56].

The literature on the effects of antioxidants on the nutritional quality of the sheep colostrum is scarce. However, melatonin implanted in ewes at 3 months of pregnancy increases fat in colostrum [57]. On the other hand, maternal antioxidant supplementation in well-fed dams during the final third of gestation has been shown to improve colostrum composition, reflected in increased concentrations of fat, protein, and immunoglobulins in small ruminants [58,59]. Therefore, difference in colostrum composition supports the need for further studies to evaluate the factors that may cause variations in colostrum composition under conditions of antioxidant supplementation in nutritionally restricted ewes.

## 5. Conclusions

Supplementation with a combination of dry grape extract (DGS) and olive-derived hydroxytyrosol (HxT) polyphenols during gestation in sheep under mild nutritional restriction, without affecting maternal body weight or condition, enhances maternal antioxidant defenses and metabolic status, as well as the birth weight and size of the lambs—particularly in more demanding pregnancies such as twins. The use of such agro-industrial by-products may offer benefits not only from an environmental perspective but also from a productive one, as promoting circular economy practices could improve performance in the most compromised gestations, such as twin pregnancies, where perinatal mortality remains one of the main limitations to sheep production worldwide. A limitation of this study is the use of a single concentration for each of the evaluated products, so further studies aiming to identify the most cost-efficient doses are foreseen. In addition, the promising results obtained under the conditions of this study should be confirmed in future experiments involving a larger number of animals under real production conditions.

## Figures and Tables

**Table 1:** Plasma concentrations (mean ± SEM) of biomarkers of metabolic status at 140 days of gestation in ewes carrying singleton and twin pregnancies with (AOx) or without (Con) antioxidants supplementation.

	Singles		Twins		Rank	AOx
Variable	Con	AOx	Con	AOx	*p*-Value	*p*-Value
BHB (mmol/L)	0.55 ± 0.04	0.64 ± 0.06	1.09 ± 0.26	0.69 ± 0.06	**0.026**	0.528
Glucose (mg/dL)	67.7 ± 3.3	69.0 ± 3.2	83.8 ± 12.7	69.5 ± 2.73	0.205	0.454
Lactate (mg/dL)	11.2 ± 1.7	14.7 ± 1.8	17.5 ± 4.4	18.40 ± 3.4	0.057	0.234
NEFAs (mmol/L)	0.55 ± 0.07	0.38± 0.04	1.15 ± 0.31	0.71 ± 0.13	**0.004**	0.227
Trig (mg/dL)	27.1 ± 1.9	29.5 ± 2.8	33.1 ± 4.2	30.6 ± 2.6	0.218	0.717
Total protein (g/dL)	7.25 ± 0.17	7.25 ± 0.23	7.20 ± 0.34	7.10 ±0.16	0.591	0.966
Urea (mg/dL)	27.2 ± 1.8	28.4 ± 3.0	31.1 ± 3.3	32.0 ± 2.3	0.117	0.500

BHB: β-hydroxybutyrate; NEFAs: non-esterified fatty acids; Trig: triglycerides.

**Table 2:** Colostrum composition (%) immediately after parturition in ewes carrying singleton and twin pregnancies with (AOx) or without (Con) antioxidants supplementation.

	Singles		Twins		Rank	AOx
Variable	Con	AOx	Con	AOx	*p*-Value	*p*-Value
**Fat** (%)	8.75 ± 2.24	11.29 ± 1.09	11.84 ± 0.06	10.41 ± 1.05	0.688	0.449
**Protein** (%)	12.87 ± 0.59	10.46 ± 1.79	11.96 ± 12.7	13.16 ± 2.87	0.597	0.752
**Lactose** (%)	3.43 ± 0.14	3.58 ± 0.21	3.43 ± 0.72	3.05 ± 0.45	0.302	0.756
**TS** (%)	27.50 ± 2.38	27.60 ± 2.10	29.31 ± 7.64	29.79 ± 3.05	0.474	0.960
**NFS** (%)	17.54 ± 0.69	15.23 ± 1.73	16.79 ± 6.70	17.50 ± 2.64	0.656	0.688

TS: total solids; NFS: non-fat solids.

**Table 3:** Biometrical traits at birth of singleton and twin lambs born to ewes carrying singleton and twin pregnancies with (AOx) or without (Con) antioxidants supplementation.

	Singles		Twins		Rank	AOx
Variable	Con	AOx	Con	AOx	*p*-Value	*p*-Value
**Nose–tail** **length** (cm)	54.3 ± 1.4	53.7 ± 2.2	53.0± 0.8	53.9 ± 1.2	0.747	0.864
**Forelimb** **length** (cm)	32.8 ± 0.6	33.0 ± 0.7	30.8± 0.6	31.3± 0.7	**0.013**	0.821
**Hindlimb** **length** (cm)	40.2 ± 1.2	40.0 ± 0.1	35.8 ± 1.2	35.7 ± 0.5	**<0.001**	0.558
**Thorax** **perimeter** (cm)	36.6 ± 1.4	37.3 ± 1.2	33.1 ± 1.0	35.3 ± 0.5	0.474	0.096
**Abdomen** **perimeter** (cm)	32.1 ± 1.8	34.3 ± 1.7	27.8 ± 1.03	31.8 ± 0.7	**0.029**	**0.038**

## Data Availability

The data presented in this study are available on request from the corresponding author. The data are not publicly available due to privacy.
